# The Causal Relationship Between Blood Lipids and Systemic Lupus Erythematosus Risk: A Bidirectional Two-Sample Mendelian Randomization Study

**DOI:** 10.3389/fgene.2022.858653

**Published:** 2022-04-13

**Authors:** Mingzhu Wang, Shuo Huang, Xiaoying Lin, Chengping Wen, Zhixing He, Lin Huang

**Affiliations:** College of Basic Medical Science, Institute of Basic Research in Clinical Medicine, Zhejiang Chinese Medical University, Hangzhou, China

**Keywords:** systemic lupus erythematosus, mendelian randomization, blood lipids, high density lipoprotein, low density lipoprotein, triglycerides, total cholesterol

## Abstract

**Background:** Although observational studies have demonstrated that blood lipids were associated with systemic lupus erythematosus (SLE), the causality of this association remains elusive as traditional observational studies were prone to confounding and reverse causality biases. Here, this study attempted to reveal the potential causal link between SLE and the levels of four blood lipids (HDL cholesterol, LDL cholesterol, TG, and TC).

**Methods:** Bidirectional two-sample Mendelian randomization (MR) was employed to explore the unconfounded causal associations between the four blood lipids and SLE. In addition, regression-based Multivariate MR (MVMR) to quantify the possible mediation effects of blood lipids on SLE. After a rigorous evaluation of the quality of studies, the single-nucleotide polymorphisms (SNPs) associated with the four blood lipids were selected from the Global Lipids Genetic Consortium (GLGC) consisted of 188,577 individuals of European ancestry, and the SNPs related to SLE were selected from a large-scale genome-wide association study (GWAS) database named IEU GWAS. Subsequently, MR analyses were conducted with inverse-variance weighted (IVW), weighted median, weighted mode, simple mode, and MR-Egger regression. Sensitivity analyses were performed to verify whether heterogeneity and pleiotropy led to bias in the MR results.

**Results:** Bidirectional two-sample MR results demonstrated that there was no significant causal association between SLE and the four blood lipids (When setting SLE as outcome, HDL cholesterol and SLE, IVW OR: 1.32, 95% CI: 1.05∼1.66, *p* = 1.78E-02; LDL cholesterol and SLE, IVW OR: 1.26, 95% CI: 1.04∼1.53, *p* = 2.04E-02; TG and SLE, IVW OR: 1.04, 95% CI: 0.71∼1.51, *p* = 8.44E-01; TC and SLE, IVW OR: 1.07, 95% CI: 0.89∼1.29, *p* = 4.42E-01; When setting SLE as exposure, SLE and HDL cholesterol, IVW OR: 1.00, 95% CI: 0.99∼1.01, *p* = 9.51E-01; SLE and LDL cholesterol, IVW OR: 0.99, 95% CI: 0.98∼1.00, *p* = 3.14E-01; SLE and TG, IVW OR: 0.99, 95% CI: 0.98∼1.00, *p* = 1.30E-02; SLE and TC, IVW OR: 0.99, 95% CI: 0.98∼1.00, *p* = 1.56E-01). Our MVMR analysis also provided little evidence that genetically determined lipid traits were significantly associated with the risk of SLE (HDL cholesterol and SLE, *p* = 9.63E-02; LDL cholesterol and SLE, *p* = 9.63E-02; TG and SLE, *p* = 8.44E-01; TC and SLE, *p* = 4.42E-01).

**Conclusion:** In conclusion, these data provide evidence that genetic changes in lipid traits are not significantly associated with SLE risk in the European population.

## Introduction

Systemic lupus erythematosus (SLE), a chronic inflammatory autoimmune disease, is characterized by the formation of autoantibodies and deposition of immune complexes. The clinical manifestations of SLE are diverse and involve multiple organs including the skin, kidneys, and joints, and undergo a chronic or recurrence and remission course (Fanouriakis et al., 2021).

Dyslipidemia is a risk factor for many diseases, such as cardiovascular disease (CVD), metabolic syndrome, and obesity (Palano et al., 2021; Alsayed et al., 2022; Subramanian, 2022). Dyslipidemia is mainly reflected by the abnormalities in low-density lipoprotein (LDL) cholesterol, high-density lipoprotein (HDL) cholesterol, triglycerides (TG), and total cholesterol (TC). In SLE patients, TC, TG, LDL cholesterol, and apolipoprotein B are usually increased, while HDL cholesterol is decreased. The above-mentioned abnormal indices are correlated with SLE disease activity (Szabó et al., 2017). Accumulating evidence indicated that SLE patients are prone to suffer from CVD (Szabó et al., 2017; Tektonidou et al., 2017). In addition, premature atherosclerosis might contribute to the high mortality of SLE patients.

Previous research had shown that blood lipids might be considered as a new element contributing to subclinical atherosclerosis in SLE patients. LDL in patients with active SLE has a stronger atherogenic effect on endothelial cells compared with the inactive or remission phase (Rodríguez-Calvo et al., 2020). Evidence suggested that high-density lipoprotein might be a new target for reducing the risk of CVD in SLE patients. Elevated levels of oxidized dysfunctional HDL and impaired cholesterol efflux were associated with atherosclerosis in SLE (Ronda et al., 2014; Smith et al., 2014). The alterations in composition and antioxidant activity induced by systemic inflammation might reduce the anti-atherosclerotic effect of HDL, leading to increased cardiovascular risk in SLE patients (Gaál et al., 2016). Besides, impaired cholesterol efflux ability was significantly associated with vascular inflammation, suggesting that treatment to improve HDL function might have a significant cardioprotective effect on SLE (Carlucci et al., 2018). Moreover, SLE patients might produce anti-lipoprotein lipase during flares, leading to decreased lipolysis and accumulation of TG-rich lipoproteins, which in turn lead to dyslipidemia that could exacerbate lupus (Bazarbashi and Miller, 2022; Ward et al., 2022). During the inactive or in remission phase, SLE patients still assume hypertriglyceridemia (Fanlo-Maresma et al., 2020). However, a research had shown that blood lipids might be considered as a new element contributing to subclinical atherosclerosis in SLE patients, whereas the relationship was not correlated with lipid concentration. Furthermore, a clinical study indicated that cardioprotective and atherogenic lipoproteins were not associated with subclinical atherosclerosis in SLE patients (Kiani et al., 2015). Therefore, the mechanism leading to premature atherosclerosis and vascular damage in SLE has not been sufficiently elucidated. Whether dyslipidemia affects SLE is still unknown. Consequently, the relationship between the blood lipids and SLE remains to be fully elucidated.

Mendelian randomization (MR) is a genetic epidemiology approach that assesses the casual association between outcomes and exposures (Li et al., 2020). MR study can get rid of the disturbance from confounders and reverse causation for employing the genetic variants as instrumental variables (IVs). As we all know, genetic variants are randomly assorted in meiosis, while disease occurs after meiosis. This classification method is consistent with the randomization of participants to experimental and control groups in randomized controlled trials. Compared with traditional observational studies, MR analysis can overcome confounding factors, loss of follow-up, time-consuming and other difficulties in conventional studies. Hence, MR analysis is more reliable and convincing.

To explore the potential causal associations between the four blood lipids and the risk of SLE, this study conducted a bidirectional two-sample MR approach by using genome-wide association study (GWAS) data from the Global Lipids Genetic Consortium (GLGC) and IEU GWAS database. The multivariable MR analysis was employed to assess the independent effects of lipids-related traits.

## Materials and Methods

### Genome-Wide Association Study Datasets

The four blood lipids GWAS summary statistics consisted of 188,577 individuals of European ancestry were obtained from the GLGC (Willer et al., 2013) (http://csg.sph.umich.edu/willer/public/lipids2013/). SLE GWASs (5,201 cases and 9,066 controls) were obtained from the IEU GWAS database, a publicly available database (https://gwas.mrcieu.ac.uk/datasets/).

### Generation of Genetic Instruments

SNPs associated with each type of blood lipid were selected as IVs (*p* < 5 × 10^–8^). Since smoking and drinking were associated with the risk of SLE, the selected SNPs suggestively related to the two above traits were removed by searching on the PhenoScanner V2 website (http://www.phenoscanner.medschl.cam.ac.uk/). We clumped the instrumental variables (*r* ^2^ < 0.01, windows size = 5000 kb) to remove the SNPs with strong linkage disequilibrium (LD), since these may cause biased results. The palindromic SNPs with a minor allele frequency (MAF) of<0.01 were excluded from the above instrument SNPs. Additionally, we selected SNPs related to SLE and applied the same method to explore whether SLE influenced HDL cholesterol, LDL cholesterol, TG, and TC. We calculated the *F* statistic to assess whether there was a weak instrumental bias. If the *F* statistic was greater than 10, it was considered that the association was strong enough so that could avoid weak instrument bias (Burgess et al., 2016).

### Removal of Horizontal Pleiotropy

MR-Pleiotropy RESidual Sum and Outlier (MR-PRESSO), a method for employing detection and correction of outliers, evaluated the potential horizontal pleiotropy. MR-PRESSO consisted of three components, including MR-PRESSO global test, MR-PRESSO outlier test and MR-PRESSO distortion test (Verbanck et al., 2018). We applied the MR-PRESSO goal test to detect the overall horizontal pleiotropy. The SNPs with *p* value less than 0.05 were removed as outlier instruments. Repeated the process until the global test was nonsignificant (*p* > 0.05). The remaining SNPs were utilized as eligible IVs to conduct the subsequent MR analysis.

### Bidirectional Two-Sample MR Analysis

Bidirectional two-sample MR methods were employed to verify the causative effect between SLE and four types of blood lipids.

Bonferroni-adjusted the results of *p* < 0.05/4 = 0.0125, which corrected the four lipid traits tested and were considered to be statistically significant. The inverse variance weighted method (IVW), the primary analyses random-effects, similar to a meta-analysis method to combine the causal effects of individual SNPs. Meanwhile, MR-Egger regression, weighted median, weighted mode, and simple mode were also used to validate the results. When blood lipid levels were considered as exposure, the effect was estimated as the odds ratio (OR) and 95% confidence intervals (CIs) of SLE risk for each SD increase in the genetically predicted lipid level. When SLE risk was treated as exposure, the resulting estimates represented the standard deviation (SD) change in lipid levels for per increase in genetic susceptibility to SLE.

### Multivariate MR Analysis

To evaluate the independent influence of the traits related to the four lipids, we performed the multivariable MR analysis. The way to obtain the multivariable MR estimate was utilized an IVW method. *p* < 0.05 for this way was considered suggestive for the potential causal association.

### Sensitivity Analysis

To rule out possible violation of the MR assumptions, multiple sensitivity analyses were performed to verify whether heterogeneity and pleiotropy of the genetic instruments have existed. Pleiotropy was the phenomenon that a single locus affects multiple phenotypes. Horizontal pleiotropy might invalidate the results of MR analysis. We used the MR-Egger regression to detect pleiotropy. The intercept term in MR-Egger regression showed no statistical difference compared with 0 (*p* > 0.05), indicating the absence of horizontal pleiotropy. Heterogeneity was quantified by Cochran Q statistic. Assuming strong heterogeneity among IVs, we used a random effects model to estimate the effect size of MR. In addition, to avoid horizontal pleiotropy caused by a single SNP, we conducted leave-one-out sensitivity analysis which was performed by sequentially discarding one SNP at a time.

All statistical analyses were performed by using the TwoSampleMR package (version 0.5.6) in R (version 4.0.5) (Gibran et al., 2018).

## Results

**FIGURE 1:**
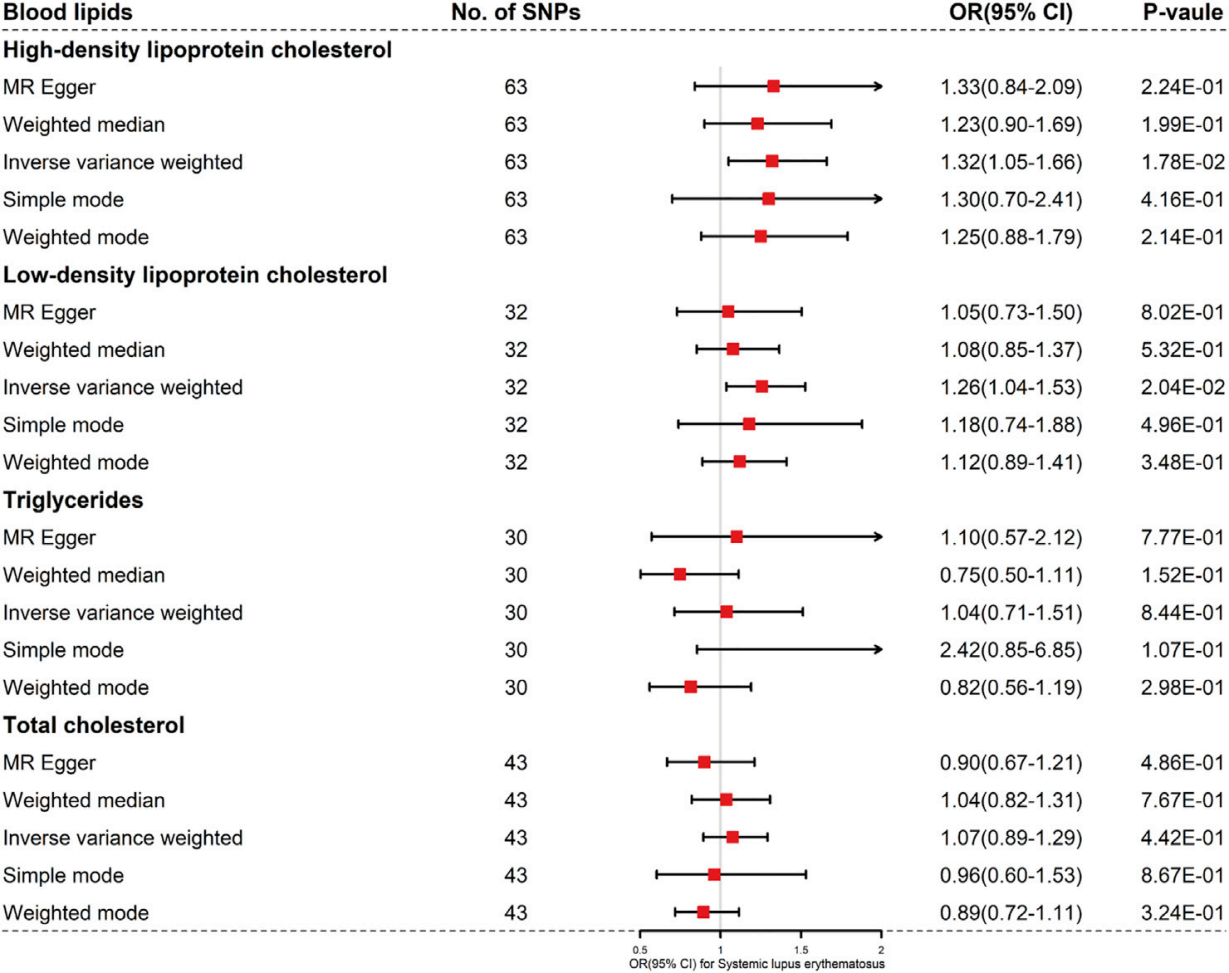
Results of the methods of MR analysis conducted to examine the relationship between blood lipids and SLE risk.

When setting SLE as the outcome, our study selected 63 HDL cholesterol, 32 LDL cholesterol, 30 TG and 43 TC genome-wide significant variants (*p* < 5 × 10^–8^) as the independent IVs (*r* ^2^ < 0.01). For these IVs, the *F*-statistics > 10 indicated that there was little chance of weak instrument variable bias (Burgess et al., 2016). Detailed information for the four exposure (LDL cholesterol, HDL cholesterol, TG and TC) was listed in Supplementary Sheet S1. Based on the results of MR-PRESSO, the outlier instrumental variables have been removed. Summary statistics for the four blood lipids were shown in Figure 1. None of the selected SNPs were associated with smoking or drinking.

**FIGURE 2:**
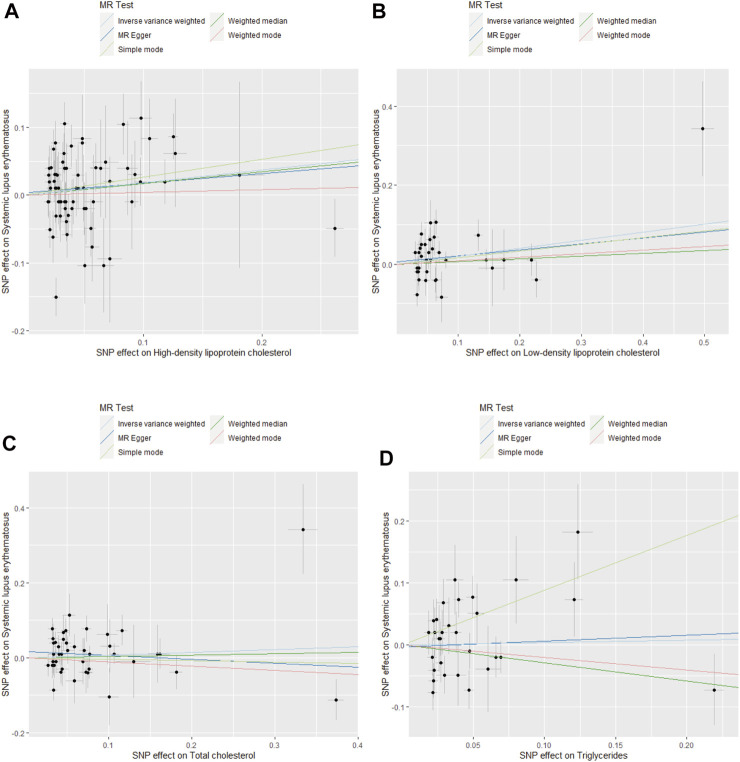
Scatter plots for MR analysis of the causal effect of blood lipids on SLE risk. **(A)** HDL cholesterol. **(B)** LDL cholesterol. **(C)** TC. **(D)** TG.

The four blood lipids were not causally associated with SLE (For HDL cholesterol, IVW OR 1.32, 95% CI 1.05–1.66, *p* = 0.018; For LDL cholesterol, IVW OR 1.26, 95%CI 1.04–1.53, *p* = 0.020; For TG, IVW OR 1.04, 95% CI 0.71–1.51, *p* = 0.844; For TC, IVW OR 1.07, 95% CI 0.89–1.29, *p* = 0.442) (Figures 1, 2 and Supplementary Figure S1 **)**.

Next, we conducted Cochran Q statistic to detect heterogeneity. There were significant heterogeneities in LDL cholesterol (I^2^ = 0.410, *p* < 0.05), HDL cholesterol (I^2^ = 0.241, *p* < 0.05), TG (I^2^ = 0.510, *p* < 0.05) and TC (I^2^ = 0.424, *p* < 0.05) (Table 1). We used a random effects model to estimate the MR effect size. The MR-Egger method suggested that there was no evidence of horizontal pleiotropy in LDL cholesterol (egger intercept = −0.003, *p* = 0.767), HDL cholesterol (egger intercept = 0.015, *p* = 0.253), TG (egger intercept = 0.017, *p* = 0.144) and TC (egger intercept = −0.022, *p* = 0.297). The plots of the leave-one-out analysis revealed that no single SNP was driving the causal link between blood lipids and SLE (Supplementary Figure S2). The funnel plots were shown in Supplementary Figure S3.

**TABLE 1:** Heterogeneity and horizontal pleiotropy analyses between SLE and blood lipids.

Exposure traits	Heterogeneity			Horizontal pleiotropy	
	Cochran Q statistic			MR-Egger	
	IVW Q	IVW *I* ^*2*^	IVW *p*	egger intercept	*p*
Low-density lipoprotein cholesterol	126.96358	0.47229	0.00001	0.00519	0.68388
High-density lipoprotein cholesterol	57.68903	0.41063	0.00681	0.00300	0.75304
Triglycerides	59.15360	0.50975	0.00079	0.01683	0.14399
Total cholesterol	72.93434	0.42414	0.00216	−0.02234	0.29687

**FIGURE 3:**
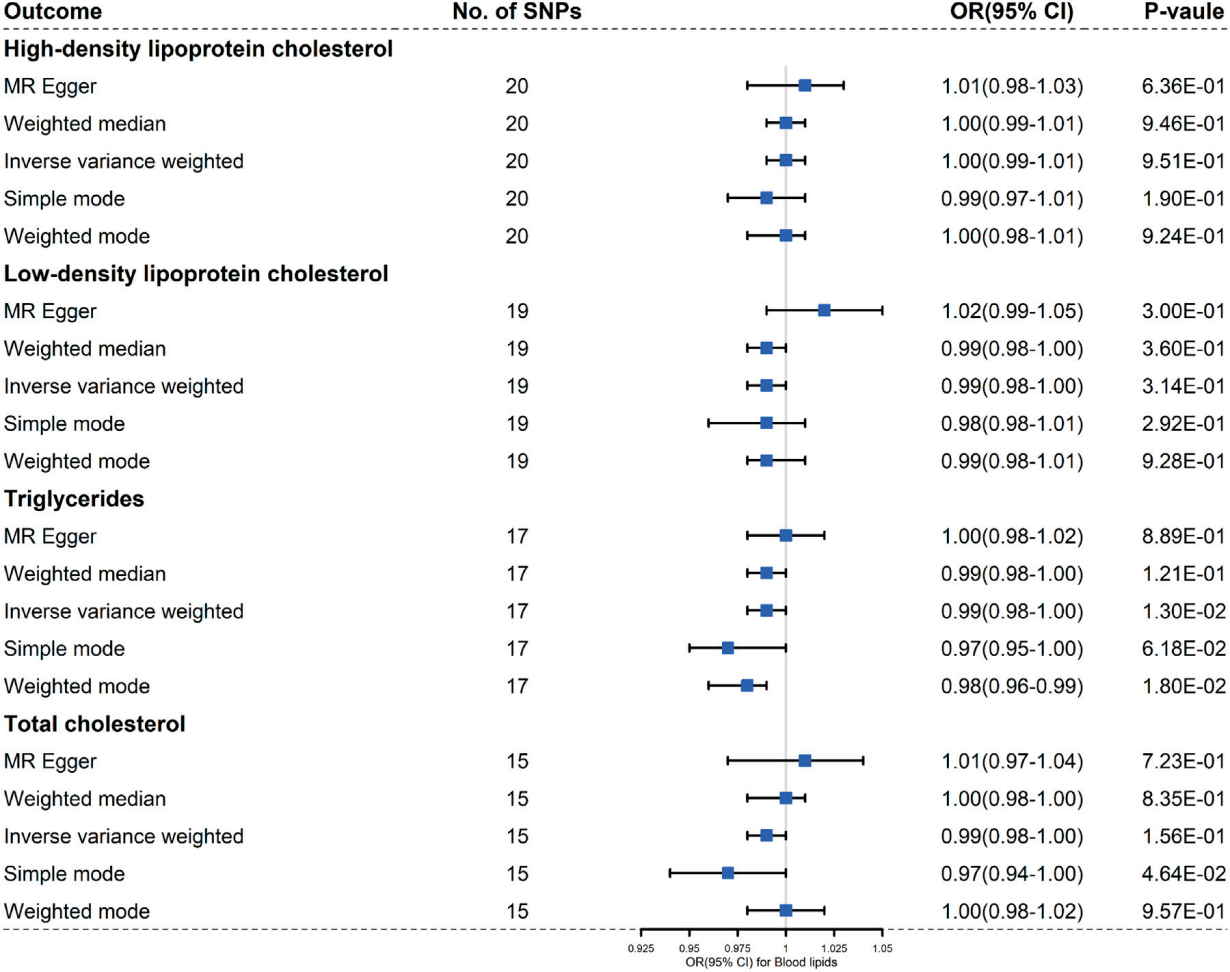
Results of the methods of MR analysis conducted to examine the relationship between SLE and blood lipids risk.

When setting SLE as exposure, we incorporated 20, 19, 17, and 15 independent and significant IVs, respectively to clarify the causal effects of SLE on HDL cholesterol, LDL cholesterol, TC and TG (Supplementary Sheet S2; Figure 3 **)**. There was no evidence suggesting that an increased risk of SLE with changes in the risk of LDL cholesterol, HDL cholesterol, TC or TG based on the different MR methods **(** Supplementary Figures S4, S5 **)**. We used MR-Egger regression to assess horizontal pleiotropy, and the results demonstrated that pleiotropy was unlikely to bias the causal relationship of HDL cholesterol (*p =* 0.253), LDL cholesterol (*p* = 0.766), TC(*p* = 0.144) and TG (*p* = 0.297) with SLE (Table 2). No horizontal pleiotropy was detected in this part.

**TABLE 2:** Heterogeneity and horizontal pleiotropy analyses between SLE and blood lipids.

Outcome	Heterogeneity			Horizontal pleiotropy	
	Cochran Q statistic			MR-Egger	
	IVW Q	IVW *I* ^2^	IVW *P*	egger intercept	*P*
High-density lipoprotein cholesterol	34.84156	0.31716	0.04737	0.01523	0.25299
Low-density lipoprotein cholesterol	57.68903	0.39727	0.00681	−0.00289	0.76609
Triglycerides	59.15360	0.04511	0.00079	0.01683	0.14399
Total cholesterol	72.93434	−0.33610	0.00216	−0.02234	0.29687

Cochran Q-value indicated that there was heterogeneity between the IVs extracted from HDL cholesterol, LDL cholesterol, TC and TG determined with the MR-Egger methods (For HDL cholesterol, *I* ^*2*^ = 0.317, *p* = 0.047; For LDL cholesterol, *I* ^*2*^ = 0.397, *p* = 0.007; For TG, *I* ^*2*^ = 0.045, *p* = 0.001; For TC, *I* ^*2*^ = −0.336, *p* = 0.002), so we used a random effects model to estimate the MR effect size (Figure 3). Due to a single SNP based on the principle that dropping one SNP at a time sequentially, leave-one-out analysis that can avoid horizontal pleiotropy was performed (Supplementary Figure S6). Forest plots and funnel plots are presented in Supplementary Figures S5, S7.

Given correlation among lipid-related characteristics, we conducted the multivariable MR analysis. The result indicated that there was no causal relationship between lipids and SLE (Table 3; Supplementary Sheet S3). With mutual adjustment for HDL cholesterol, LDL cholesterol, TG and TC, the association between LDL cholesterol and risk of SLE was no-significance (OR 1.51, 95% CI 0.48–4.69, *p* = 0.48). Also, the result showed that genetically predicted HDL cholesterol, TG and TC were not associated with risk of SLE (Table 3).

**TABLE 3:** Multivariable MR analysis between SLE and blood lipids.

Exposure	Outcome	No. of SNPs	Beta	SE	*p*-vaule
HDL cholesterol	Systemic lupus erythematosus	75	0.410	0.579	0.478
LDL cholesterol		64	0.792	1.102	0.472
Total cholesterol		72	−1.071	1.319	0.417
Triglycerides		38	0.410	0.527	0.436

## Discussion

It is well known that SLE patients had a high risk of suffering from CVD and atherosclerosis (AS) (Wang et al., 2022). Dyslipidemia was one of the typical hallmarks of AS and CVD, characterized by the elevated LDL cholesterol, TG and TC, and reduced HDL cholesterol. Dyslipidemia was observed at early diagnosis of lupus and correlated with the disease activity of SLE (Szabo et al., 2017; Robinson et al., 2022). This study aimed to illustrate the causal relationship between SLE and blood lipids (LDL cholesterol, HDL cholesterol, TG and TC) using two sample and multivariable MR analysis. However, no causal association was demonstrated between HDL cholesterol, LDL cholesterol, TG and TC and SLE after eliminating the complex confounding factors. Our MVMR analysis provided similar results, with no genetic evidence that lipid traits were significantly associated with SLE risk. Therefore, the summary of the above results found no causal relationship between dyslipidemia and SLE, excluding complex confounding factors. This demonstrated that these four lipids do not contribute to the risk of SLE at the genetic level excluding other factors.

In fact, the possible role of genetic characteristics in dyslipidemia and CVD has not been well defined in SLE patients. Recently, previous studies have indicated that the susceptibility of SLE patients to atherosclerosis and CVD was not entirely attributable to traditional risk factors, and chronic inflammatory state exists in patients with high disease activity and long disease course, which enlarge the incidence of cardiovascular events (Skaggs et al., 2012; Arvind et al., 2016). Inflammation and enhanced oxidative stress have been shown to be basic risk factors for the onset and progression of CVD (Mirmiran et al., 2022). The increase in CVD risk in SLE patients is not judged by traditional risk factors alone, and chronic inflammation may also play a role, such as elevated triglyceride-rich lipoprotein leading to low-grade inflammation (Bazarbashi and Miller, 2022); changes in composition and antioxidant activity reducing the anti-atherosclerotic effect of HDL which lead to increased cardiovascular risk in SLE patients (Gaal et al., 2016).

Consistent with this, a new clinical study indicated that anti-dsDNA positively might directly influence the development of CVD in SLE patients by modulating inflammation and clot-related molecules and regulating partially induced endothelial cell activation. In addition, inflammatory cascades may play an important role in the susceptibility of rheumatic diseases to cardiovascular disease. The mechanism may be elevated levels of oxidized lipids, such as oxidized LDL and pro-inflammatory HDL, leading to an inflammatory cascade that eventually leads to plaque formation (Sagar et al., 2020). Furthermore, a clinical study demonstrated that LDL in patients with active SLE has a stronger atherogenic effect on endothelial cells in the inactive or in remission phase compared with LDL in the same patients. It indicated that the relationship between SLE and lipids may also be related to the stage of SLE.

In addition, the parameters related to increased cardiovascular risk were associated with the presence and titer of anti-dsDNA antibodies, and the relationship was independent of the other cardiovascular risk factors (Patiño-Trives et al., 2021). Certainly, glucocorticoid exposure may also play an important role in CVDs, for example, the high-dose of prednisolone promote carotid intima-media thickness progression in SLE (Ajeganova et al., 2020; Lengton et al., 2021). It is still noteworthy that the use of glucocorticoid in SLE should be deserved attention due to the side effects of glucocorticoid in the cardiovascular risk.

Some limitations of our analysis need to be considered. Firstly, the summary-level statistics did not allow us to conduct a hierarchical analysis of covariates adjusted by the original GWAS. Secondly, in our MR methods, linear regression models were performed to assume the relationship between blood lipids and SLE, because the summary statistics did not allow us to explore the non-linear association between blood lipids and SLE. While linearity could be viewed as a first-order approximation of any nonlinear relationship, the simple assumption of linearity is not always justified in practice (Thompson et al., 2014). Another notable weakness was that the findings may not be applicable to non-European ancestry populations, as the genetic IVs were extracted from GWAS database of European ancestry participants.

## Conclusion

Taken together, the study suggested none of the lipid traits was associated with SLE risks. More work is needed to confirm the potential link between lipid properties and SLE risk.

## Data Availability

Publicly available datasets were analyzed in this study. This data can be found here: http://csg.sph.umich.edu/willer/public/lipids2013/ https://gwas.mrcieu.ac.uk/datasets/.

## Supplementary Material

Supplementary Figure S1 Forest plots of the causal effect of blood lipids on SLE risk. **(A)** HDL cholesterol. **(B)** LDL cholesterol. **(C)** TC. **(D)** TG.

Supplementary Figure S2 MR leave−one−out sensitivity analysis for blood lipids on SLE risk. **(A)** HDL cholesterol. **(B)** LDL cholesterol. **(C)** TC. **(D)** TG.

Supplementary Figure S3 Funnel plots of the causal effect of blood lipids on SLE risk. **(A)** HDL cholesterol. **(B)** LDL cholesterol. **(C)** TC. **(D)** TG.

Supplementary Figure S4 Scatter plots of the causal effect of SLE on bloods risk. **(A)** HDL cholesterol. **(B)** LDL cholesterol. **(C)** TC. **(D)** TG.

Supplementary Figure S5 Forest plots of the causal effect of SLE lipids on bloods risk. **(A)** HDL cholesterol. **(B)** LDL cholesterol. **(C)** TC. **(D)** TG.

Supplementary Figure S6 MR leave−one−out sensitivity analysis for SLE on blood lipids risk. **(A)** HDL cholesterol. **(B)** LDL cholesterol. **(C)** TC. **(D)** TG.

Supplementary Figure S7 Funnel plots of the causal effect of SLE on blood lipids risk. **(A)** HDL cholesterol. **(B)** LDL cholesterol. **(C)** TC. (**D)** TG.
