# Supplementary figures and images for: The Causal Relationship Between Blood Lipids and Systemic Lupus Erythematosus Risk: A Bidirectional Two-Sample Mendelian Randomization Study
Source: Front Genet. 2022 Apr 13;13:858653. doi: 10.3389/fgene.2022.858653 (PMC9043646; doi:10.3389/fgene.2022.858653)

**a**

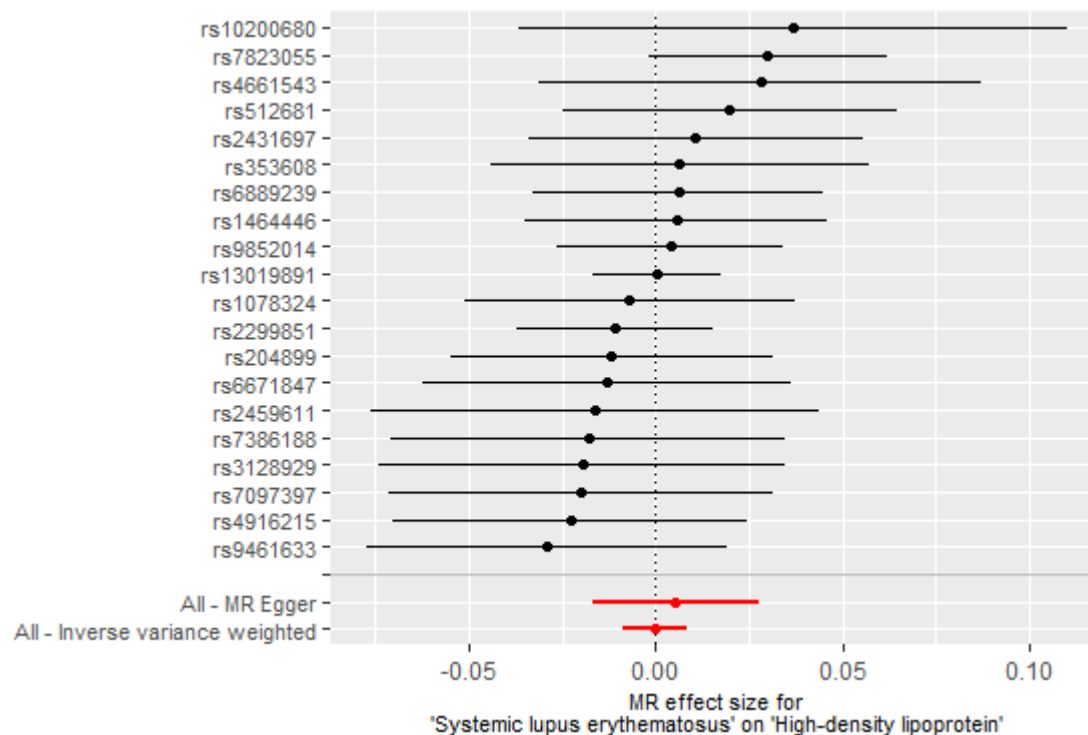

**b**

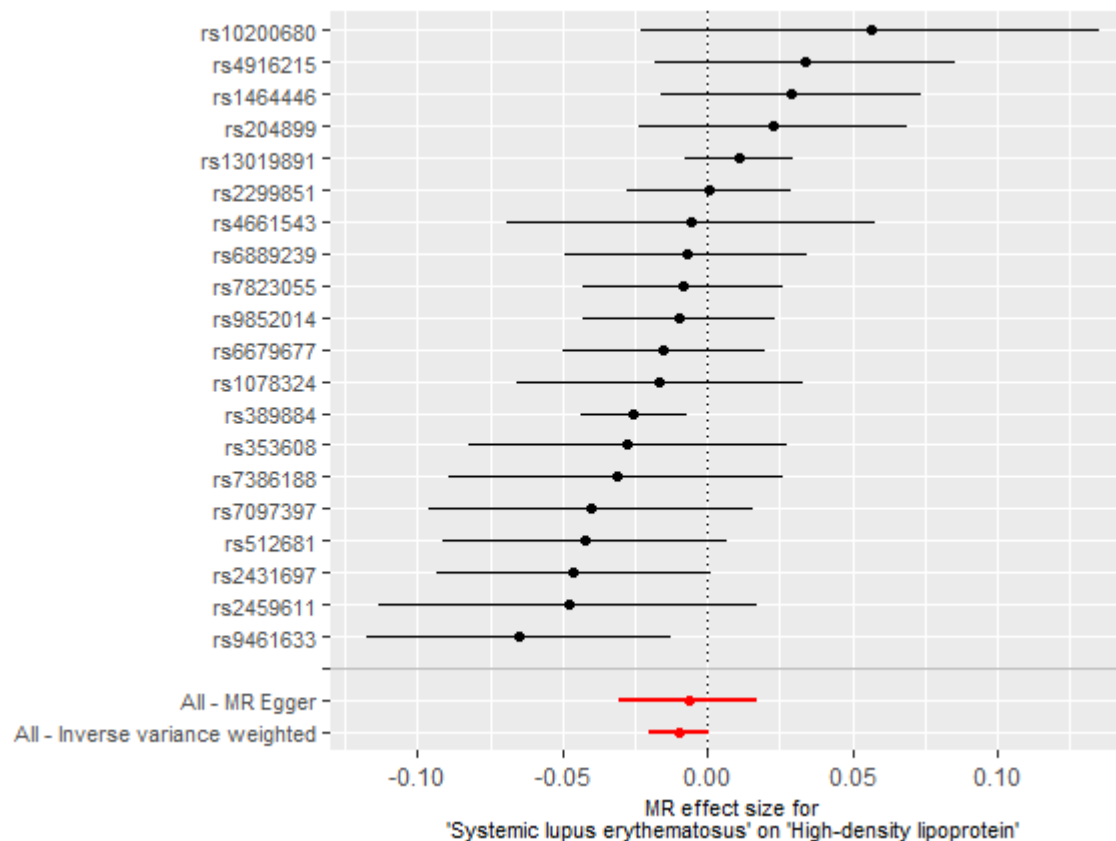

c

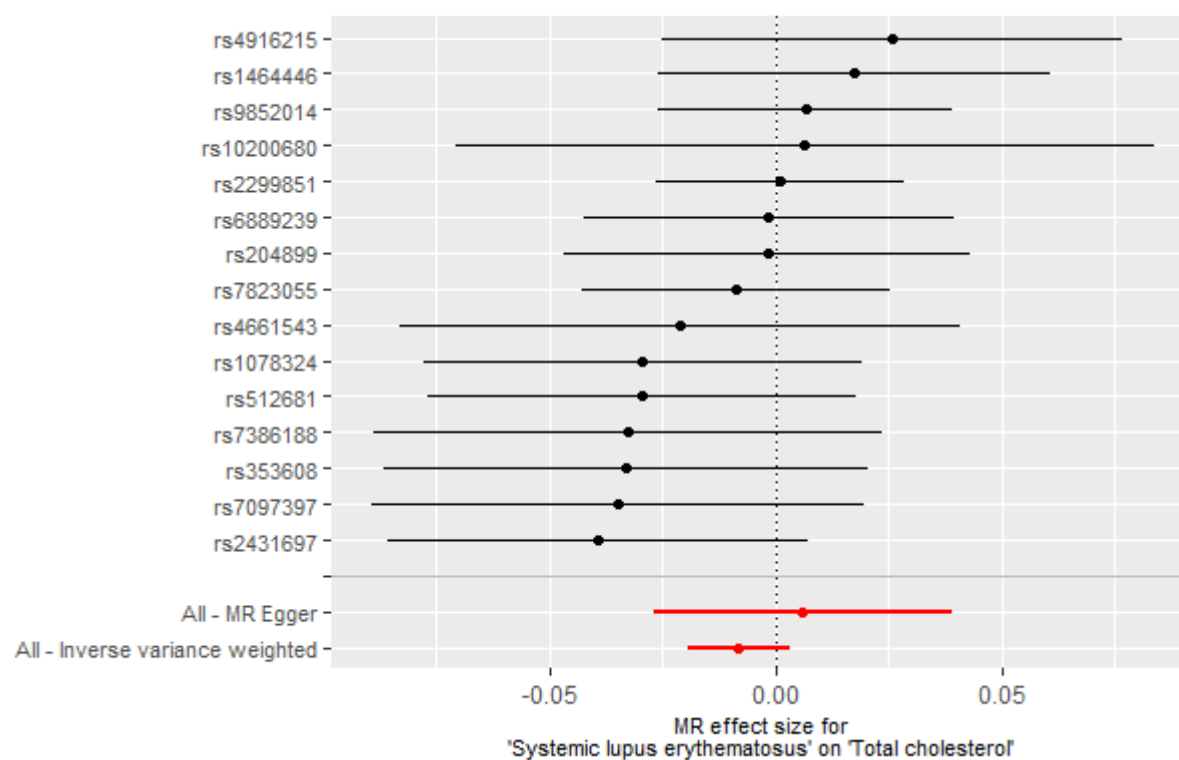

d

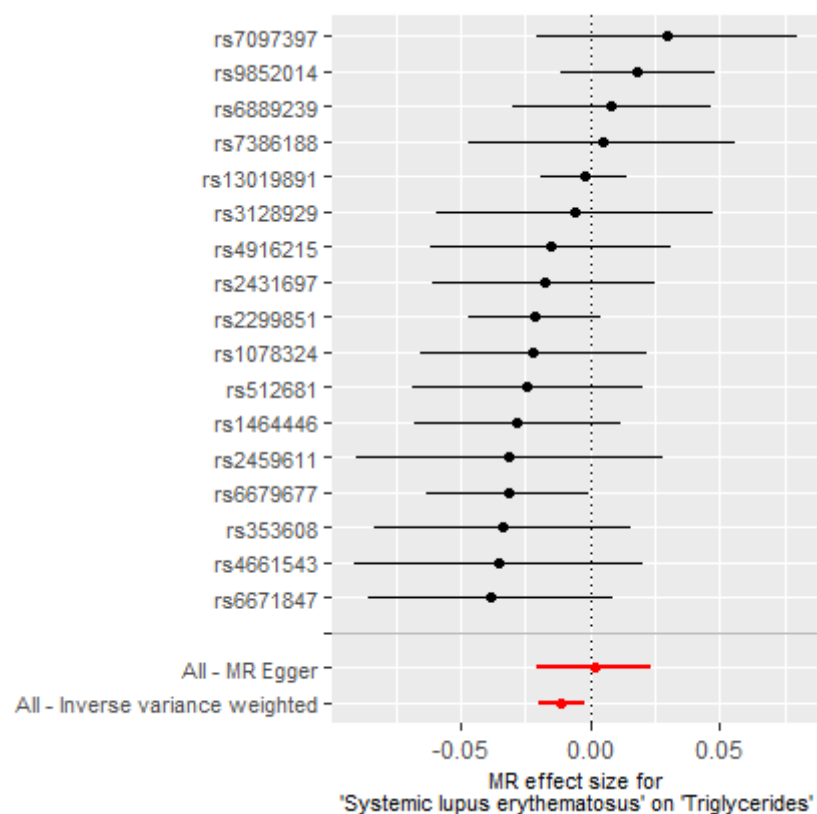

Supplement: Supplementary file 1 [file Image5.pdf]

**a**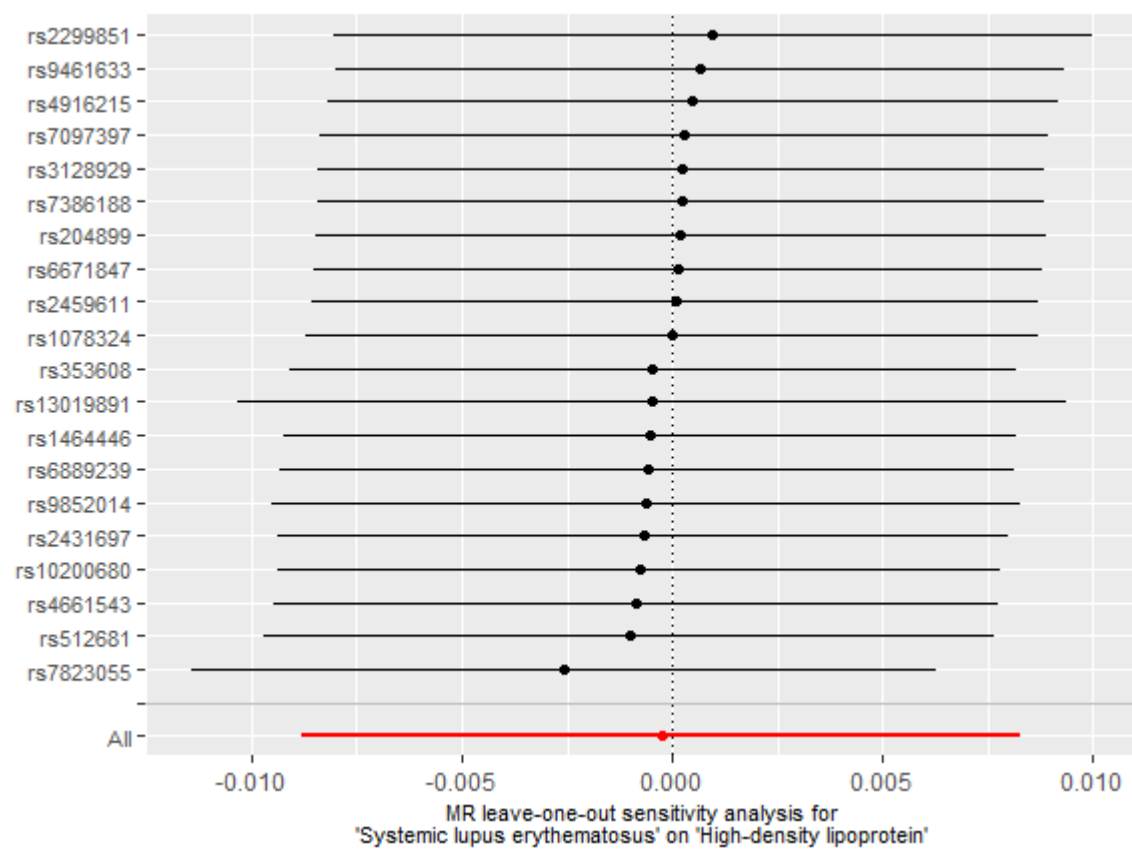**b**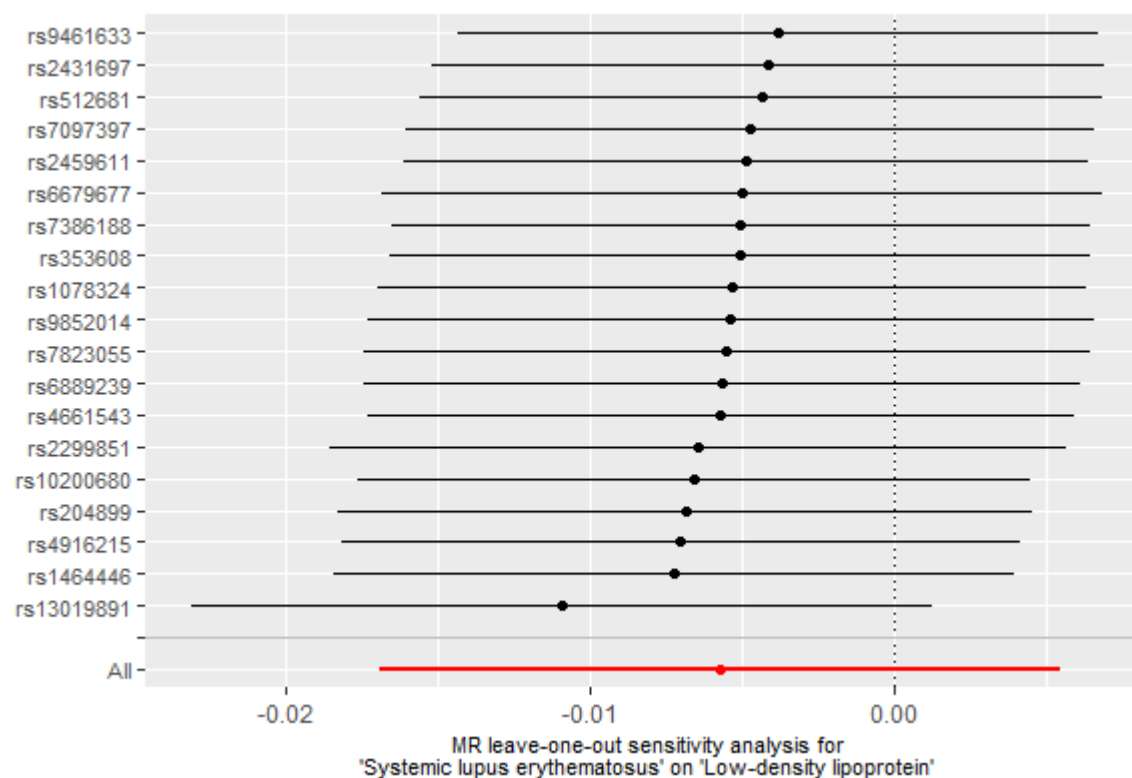

c

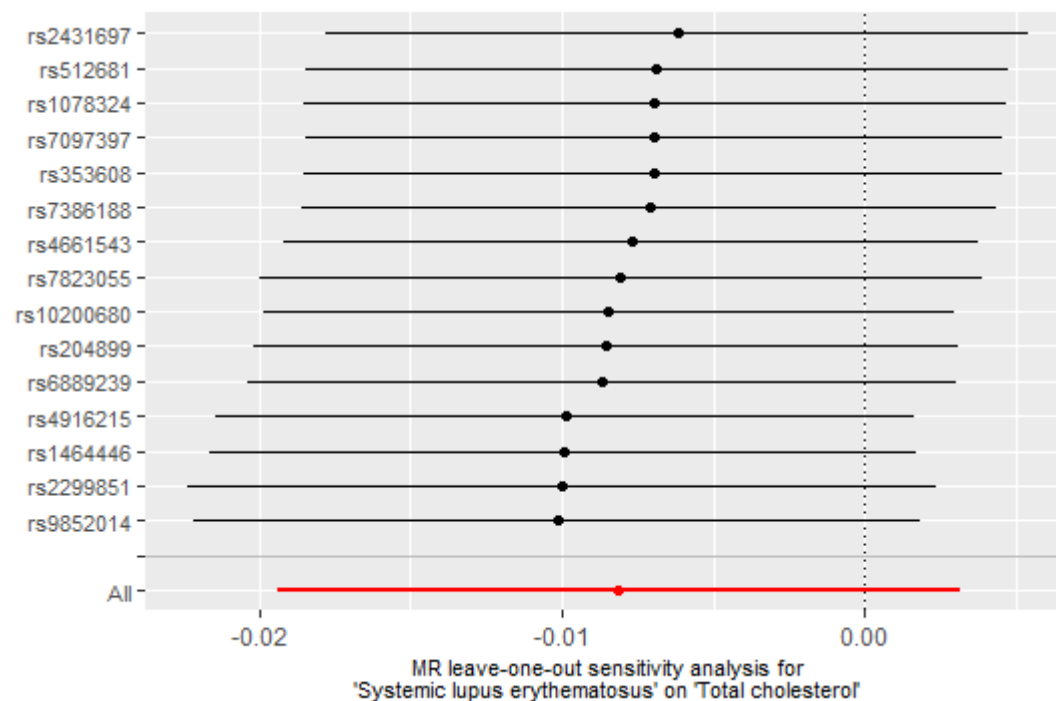

d

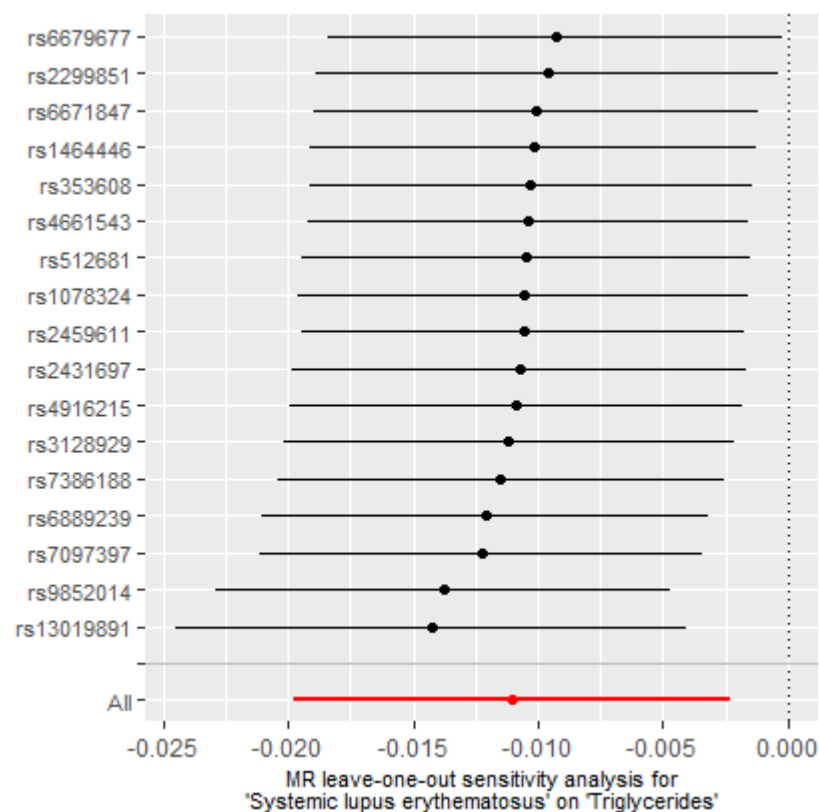

Supplement: Supplementary file 2 [file Image6.pdf]

a

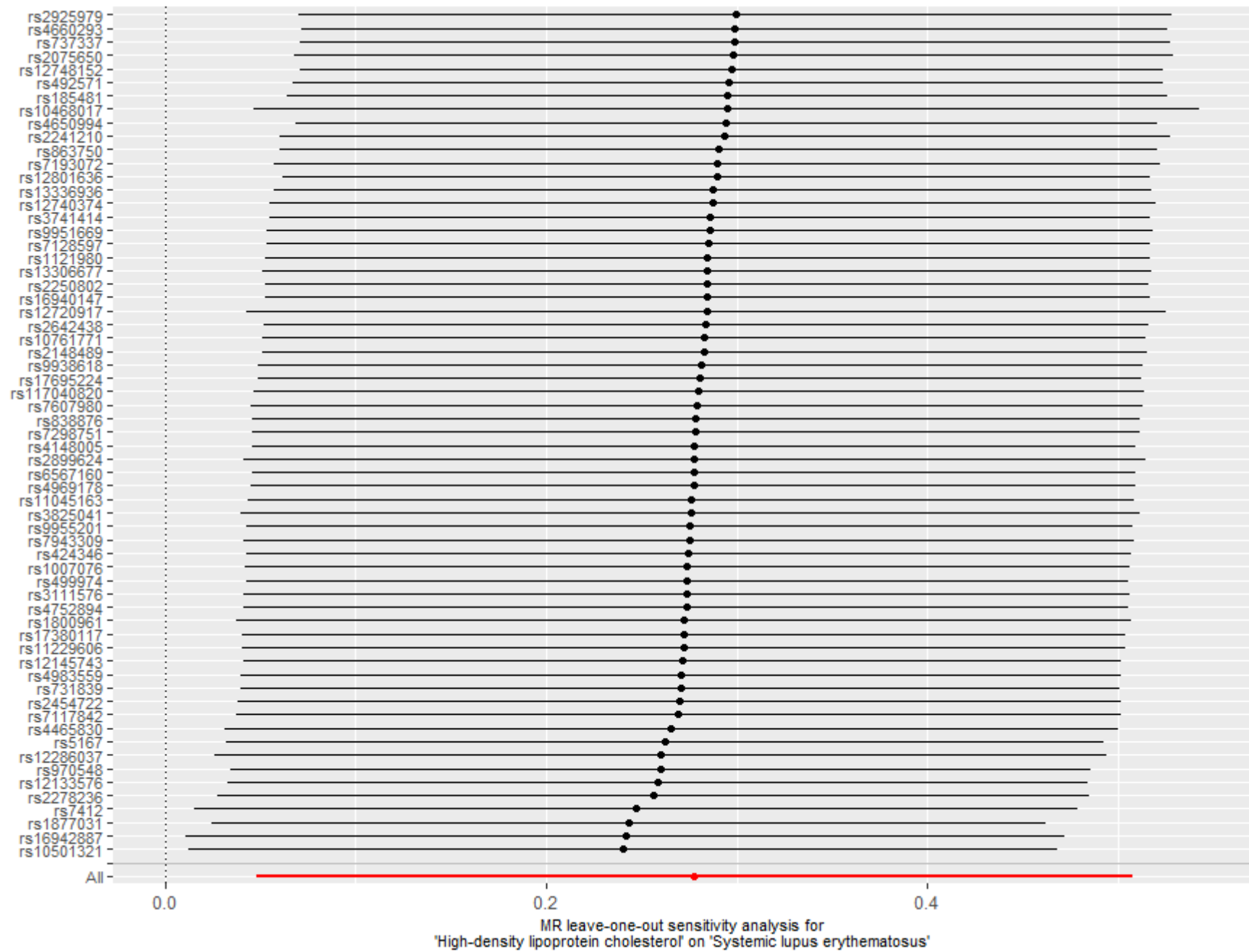

**b**

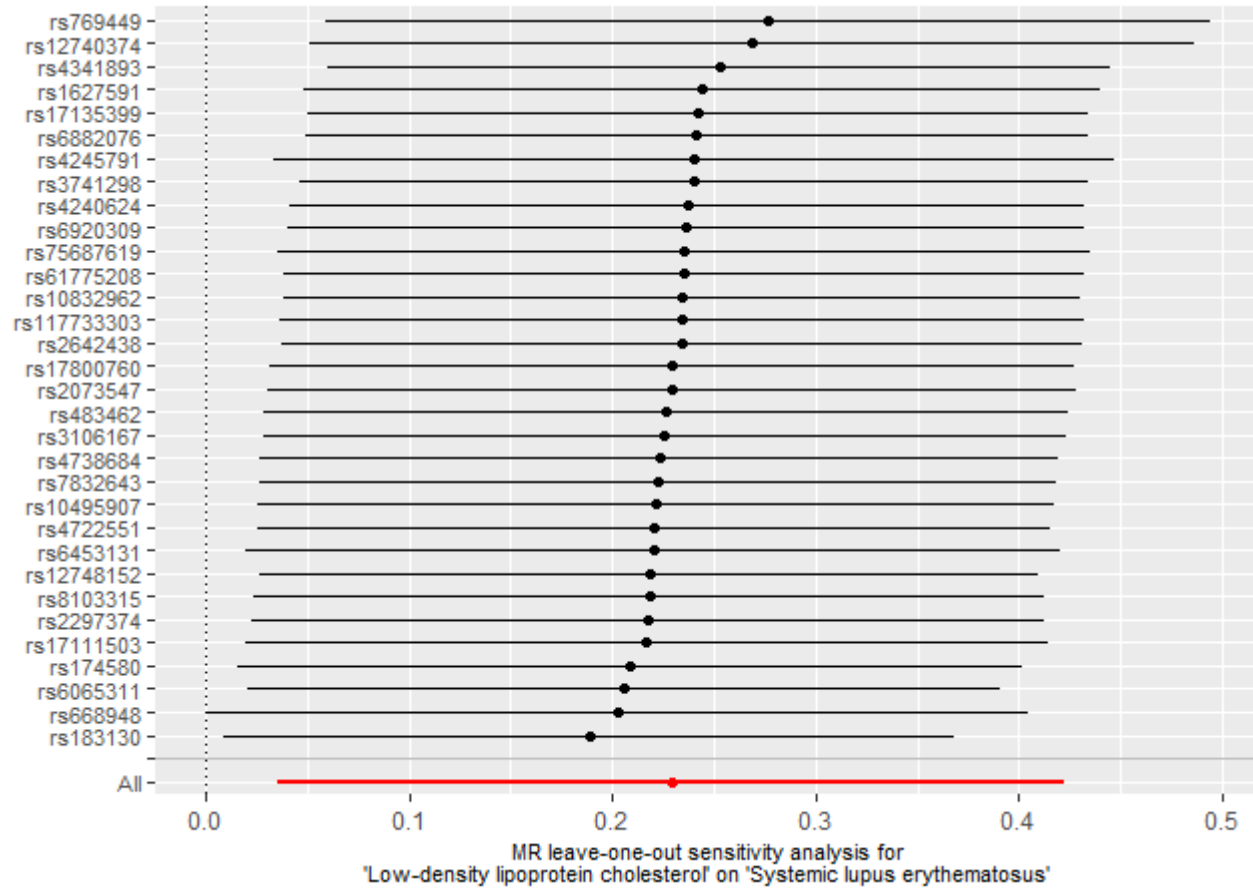

**c**

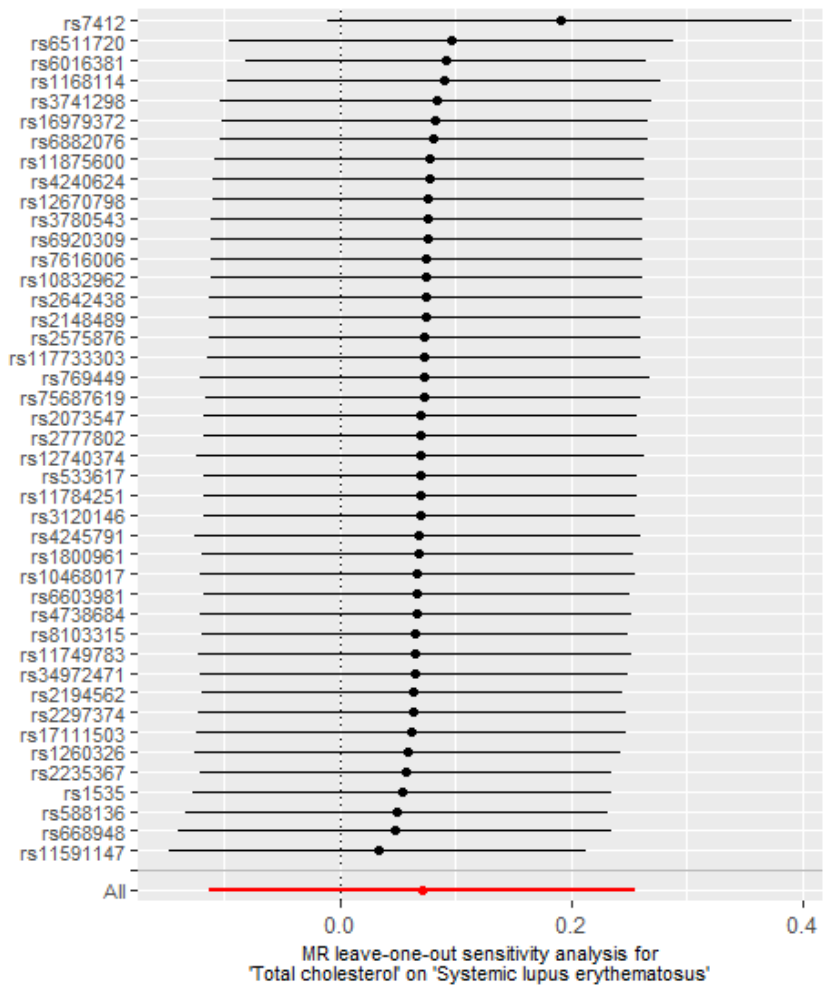

**d**

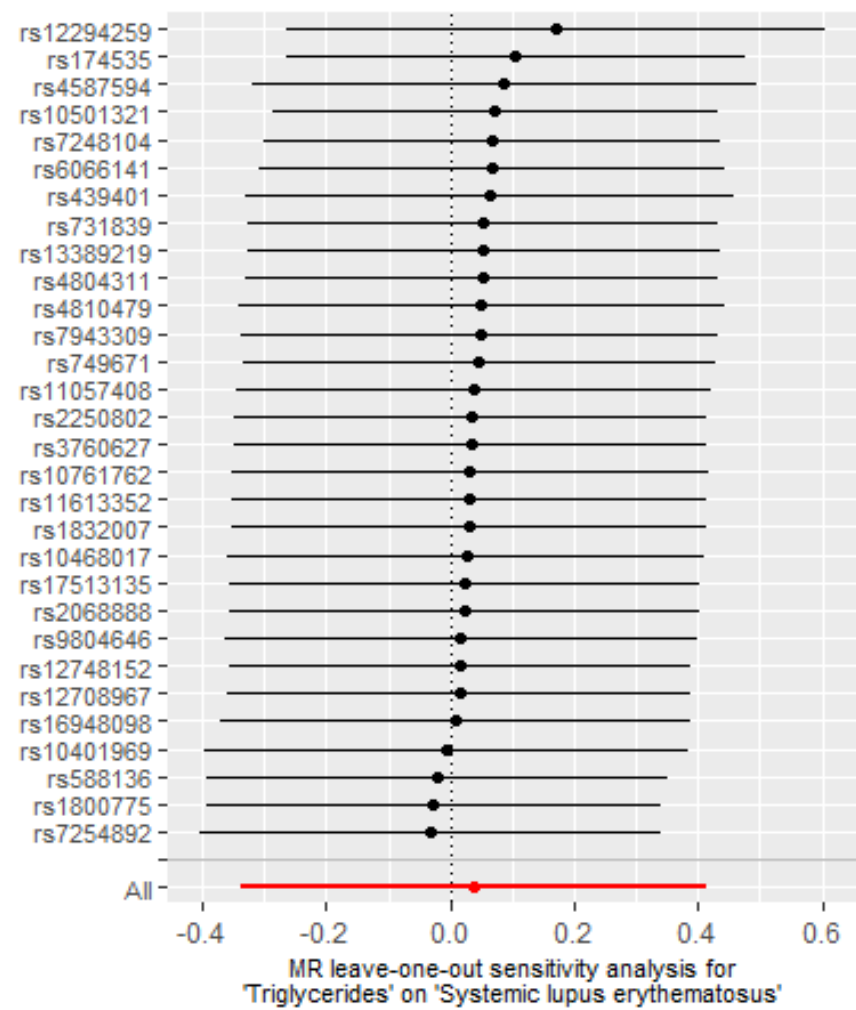

Supplement: Supplementary file 4 [file Image2.pdf]

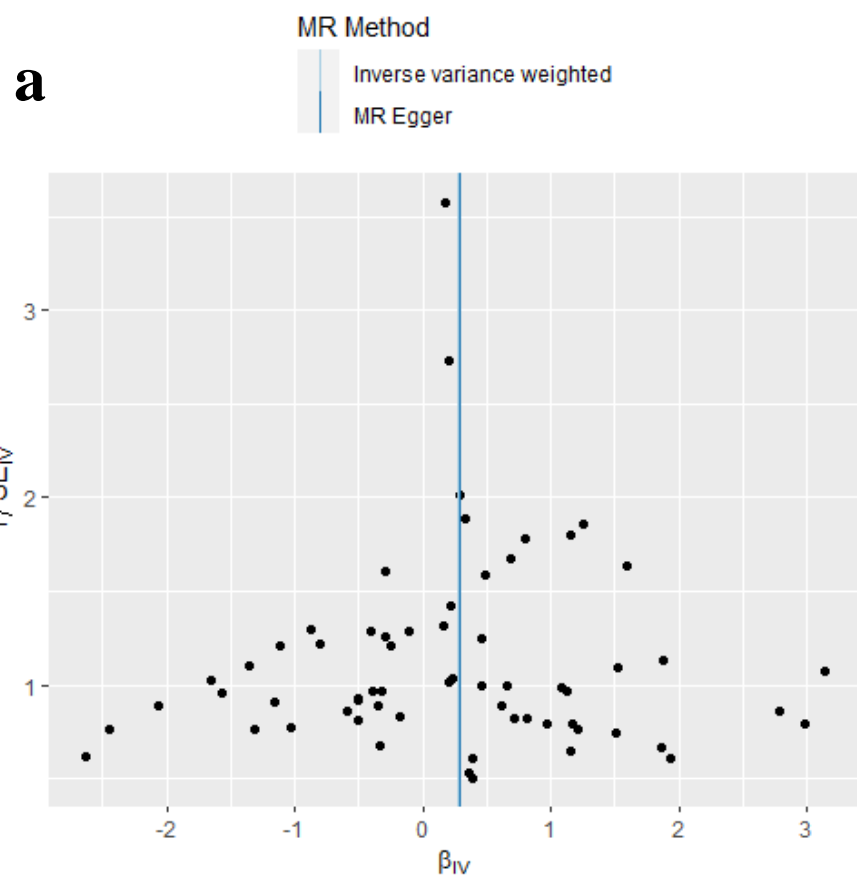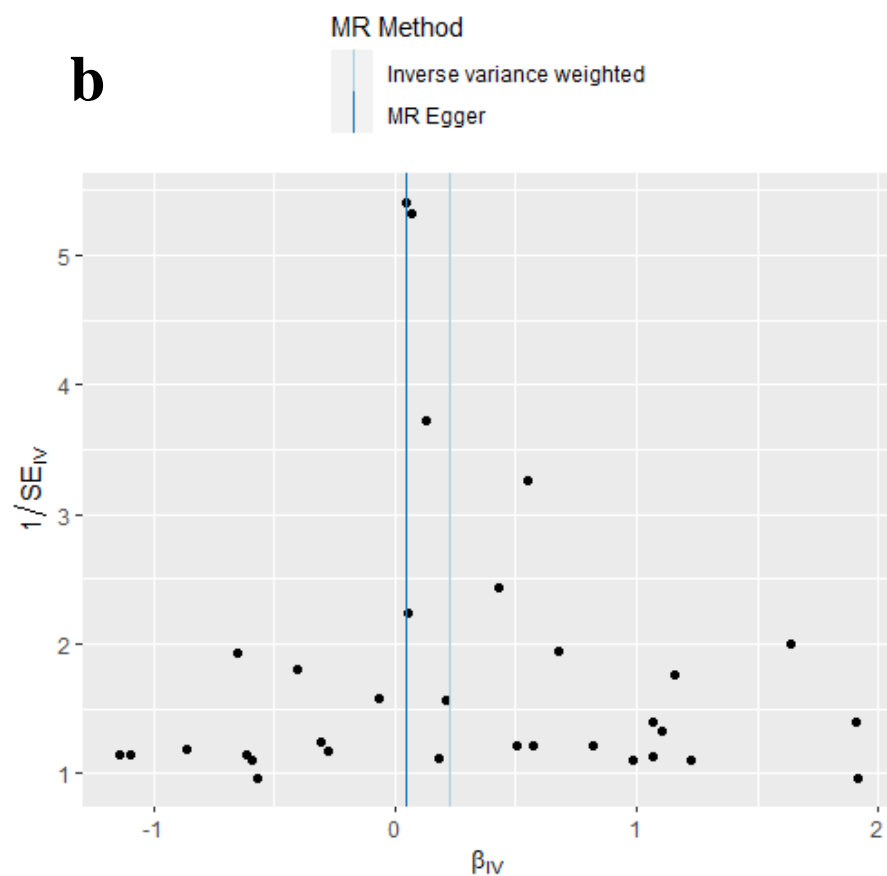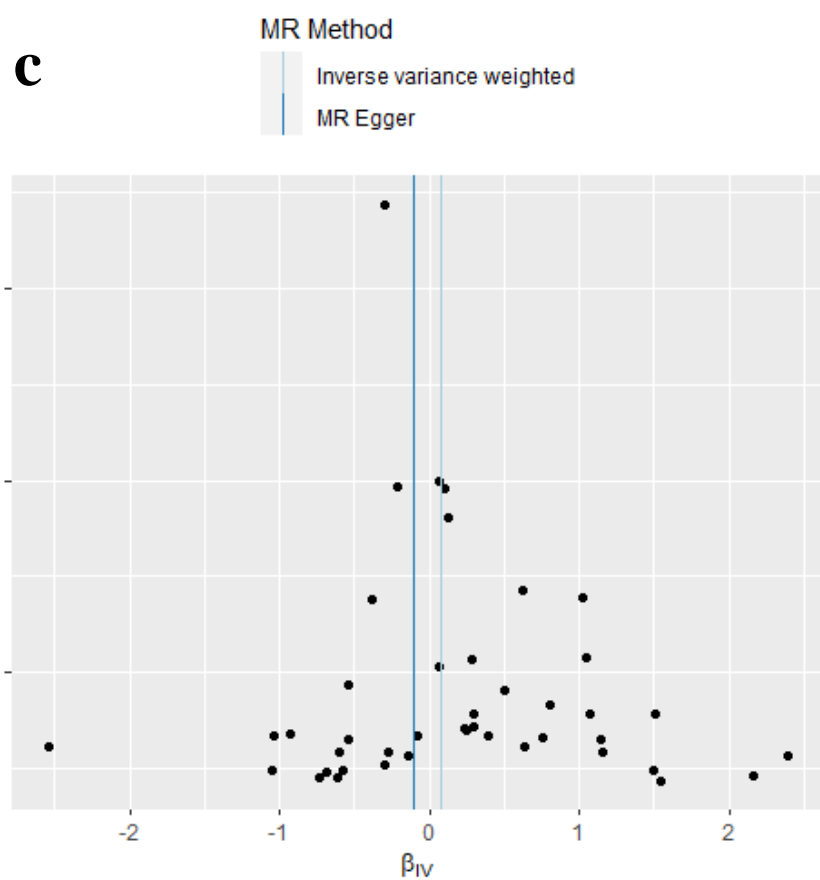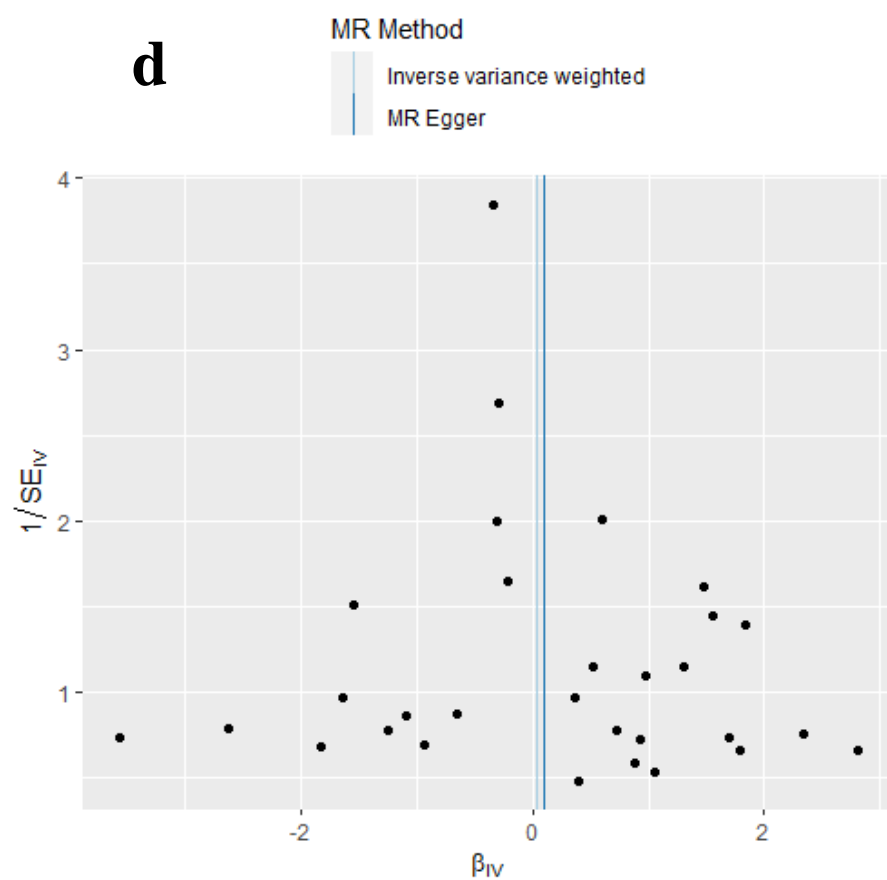

Supplement: Supplementary file 5 [file Image3.pdf]

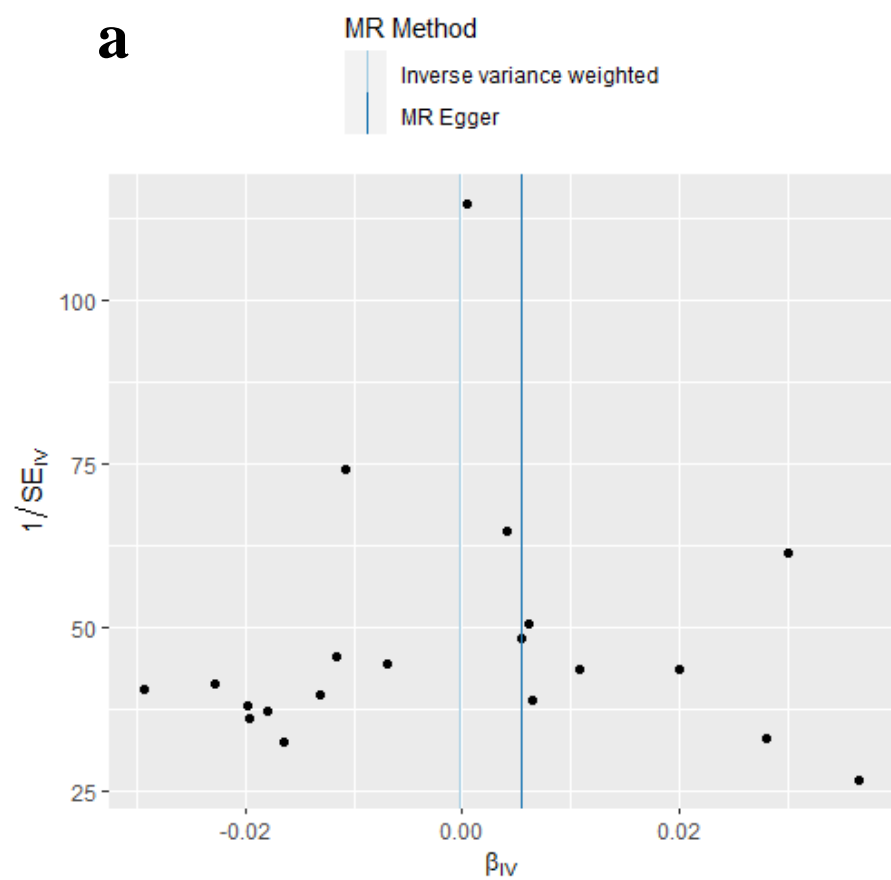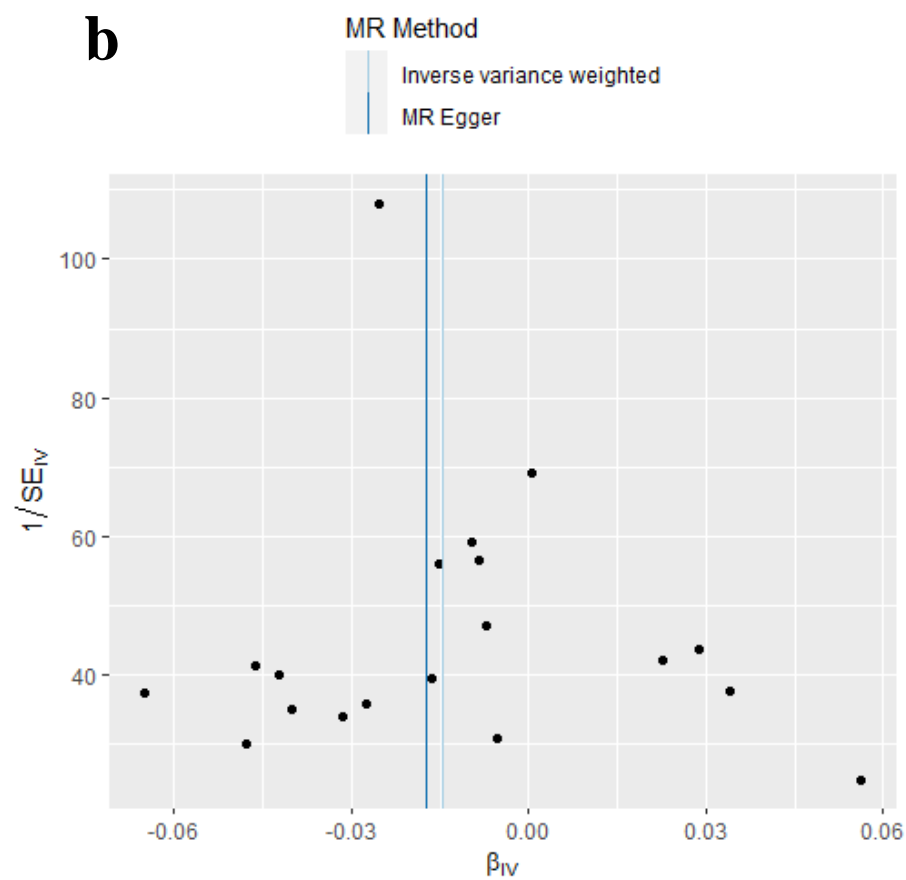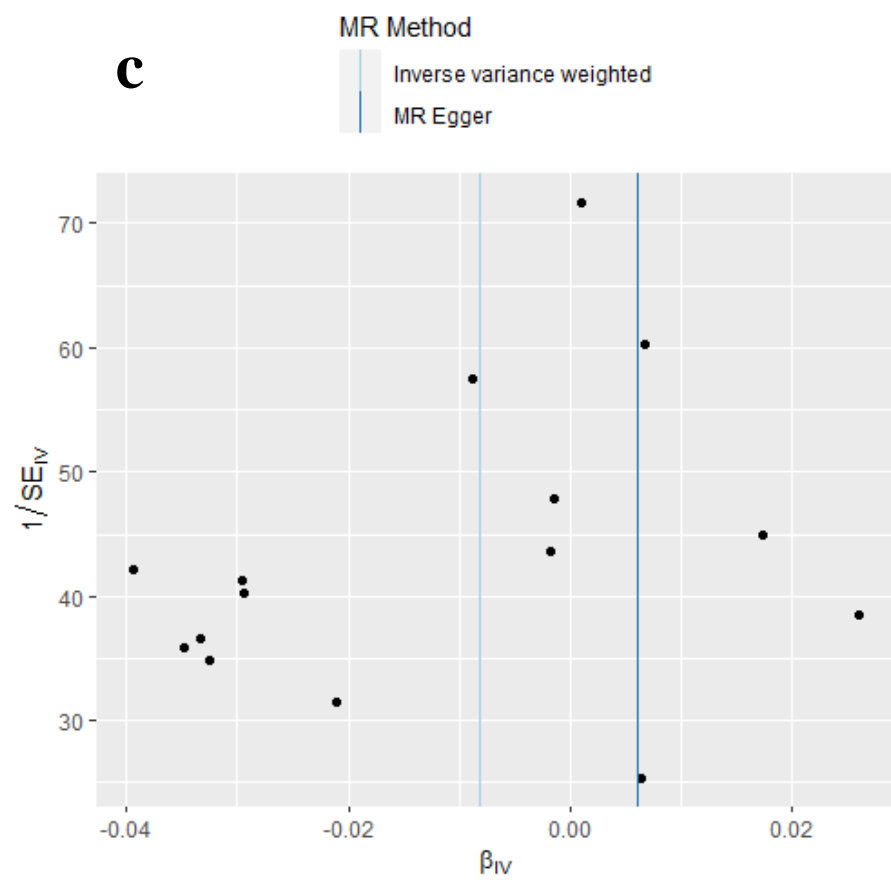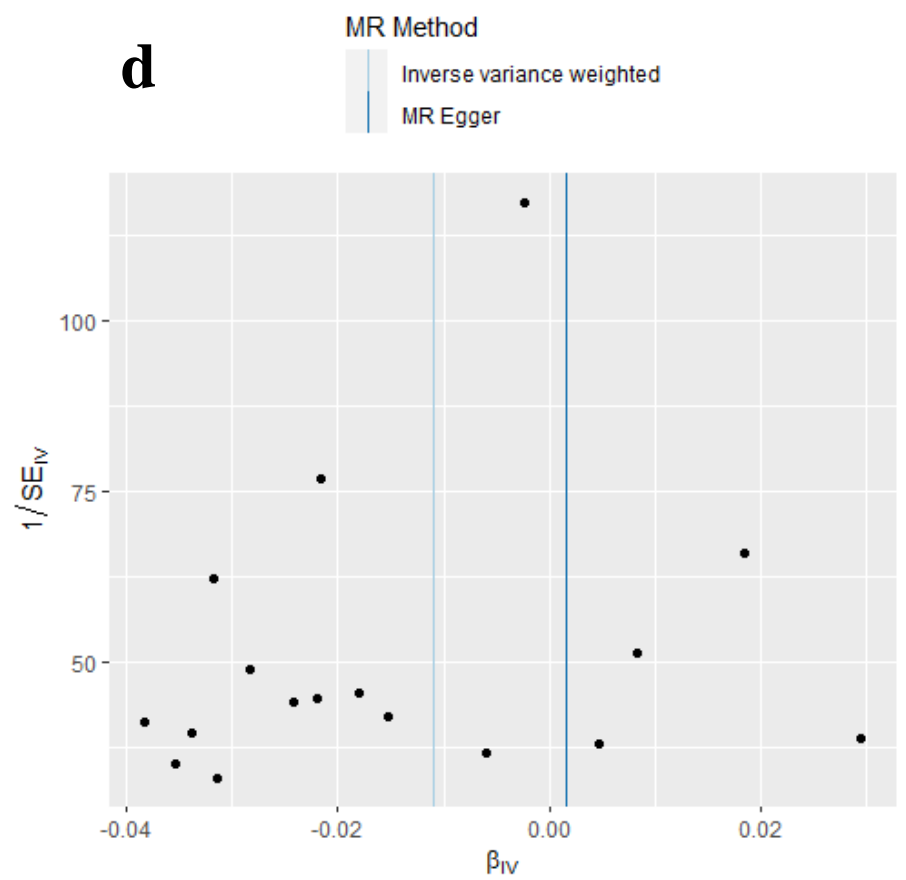

Supplement: Supplementary file 7 [file Image7.pdf]

a

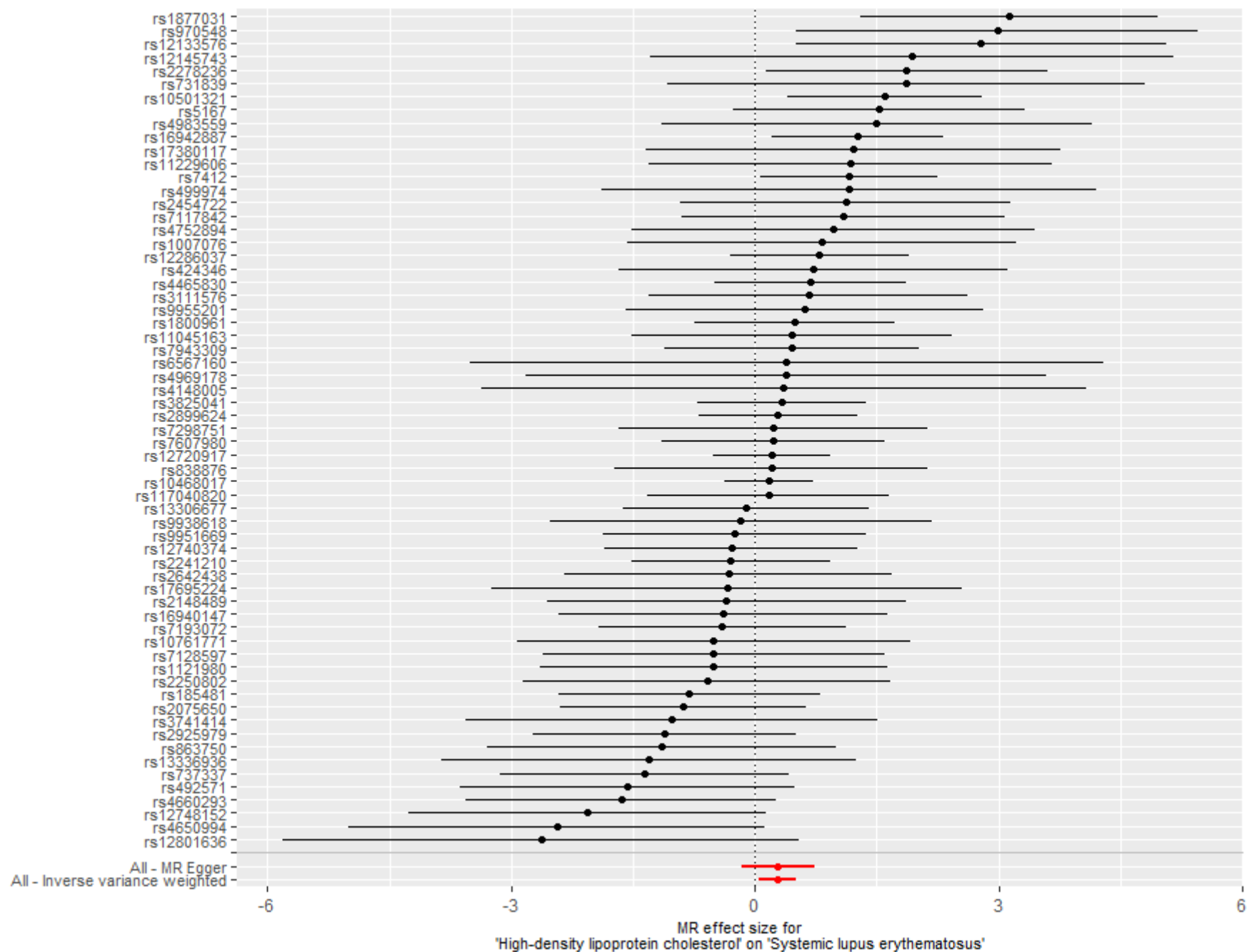

b

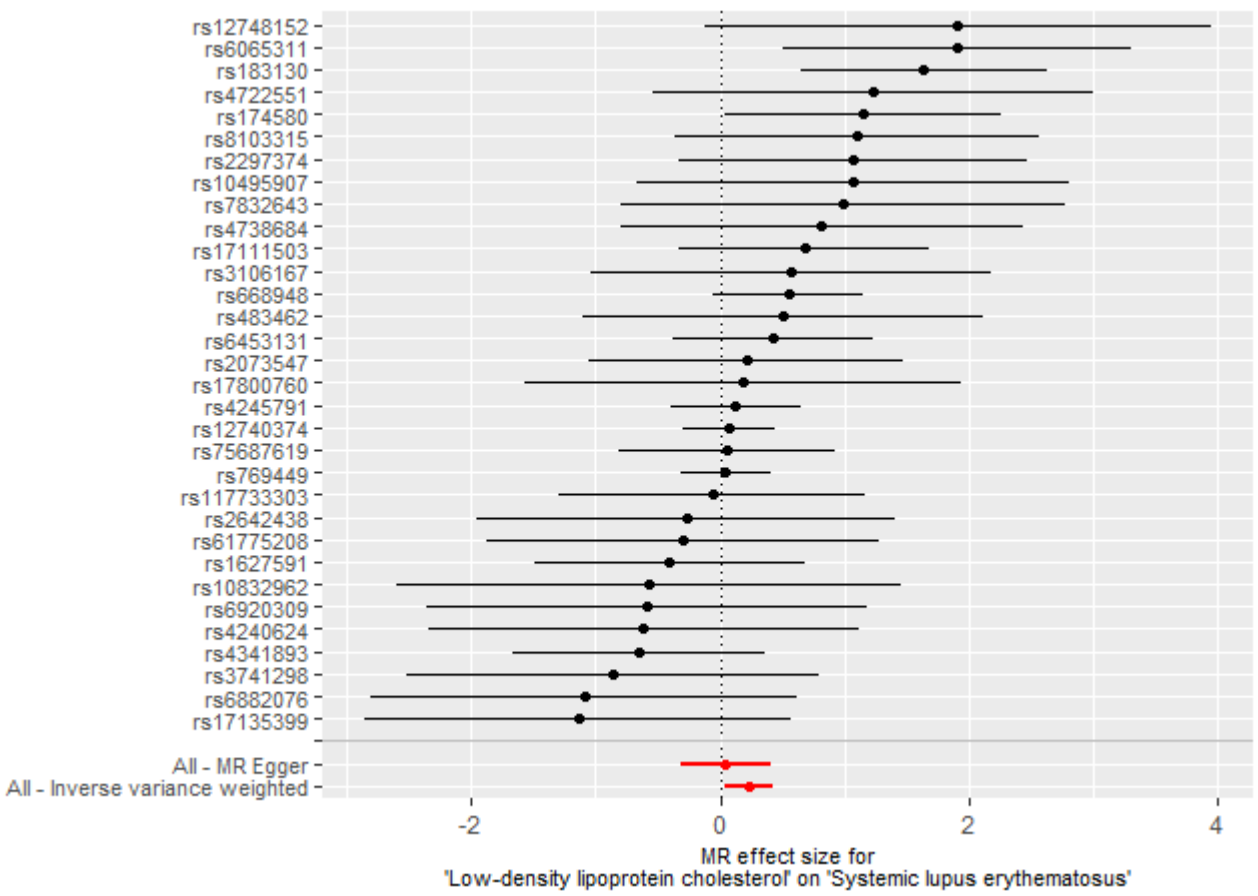

c

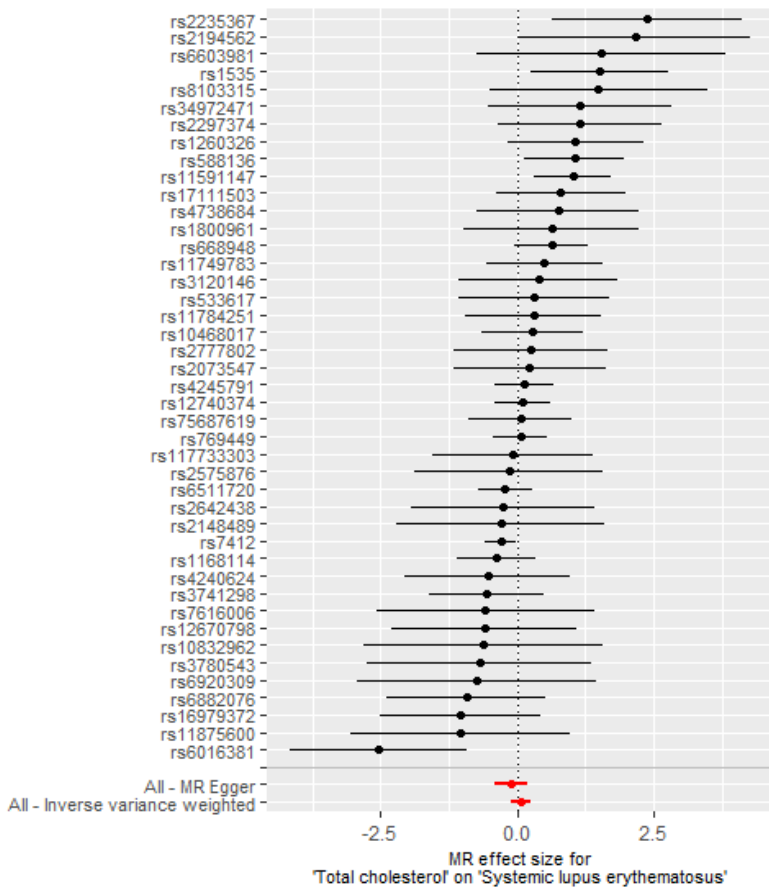

d

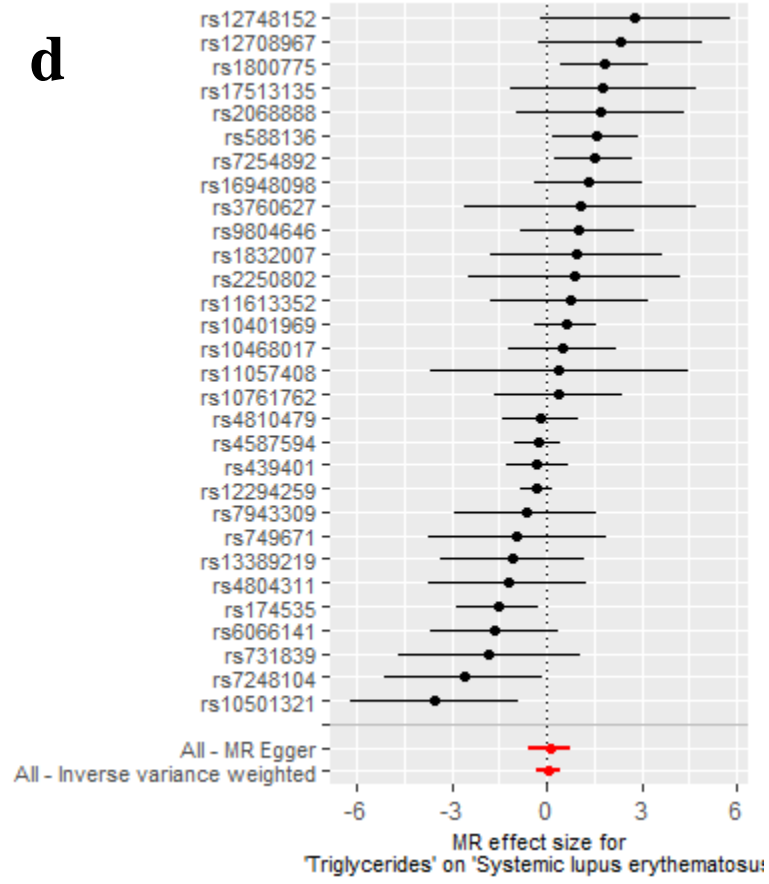

Supplement: Supplementary file 8 [file Image1.pdf]
