# Supplementary material for: The Causal Relationship Between Blood Lipids and Systemic Lupus Erythematosus Risk: A Bidirectional Two-Sample Mendelian Randomization Study
Source: Front Genet. 2022 Apr 13;13:858653. doi: 10.3389/fgene.2022.858653 (PMC9043646; doi:10.3389/fgene.2022.858653)
Supplement: Supplementary file 3 [file Image4.pdf]

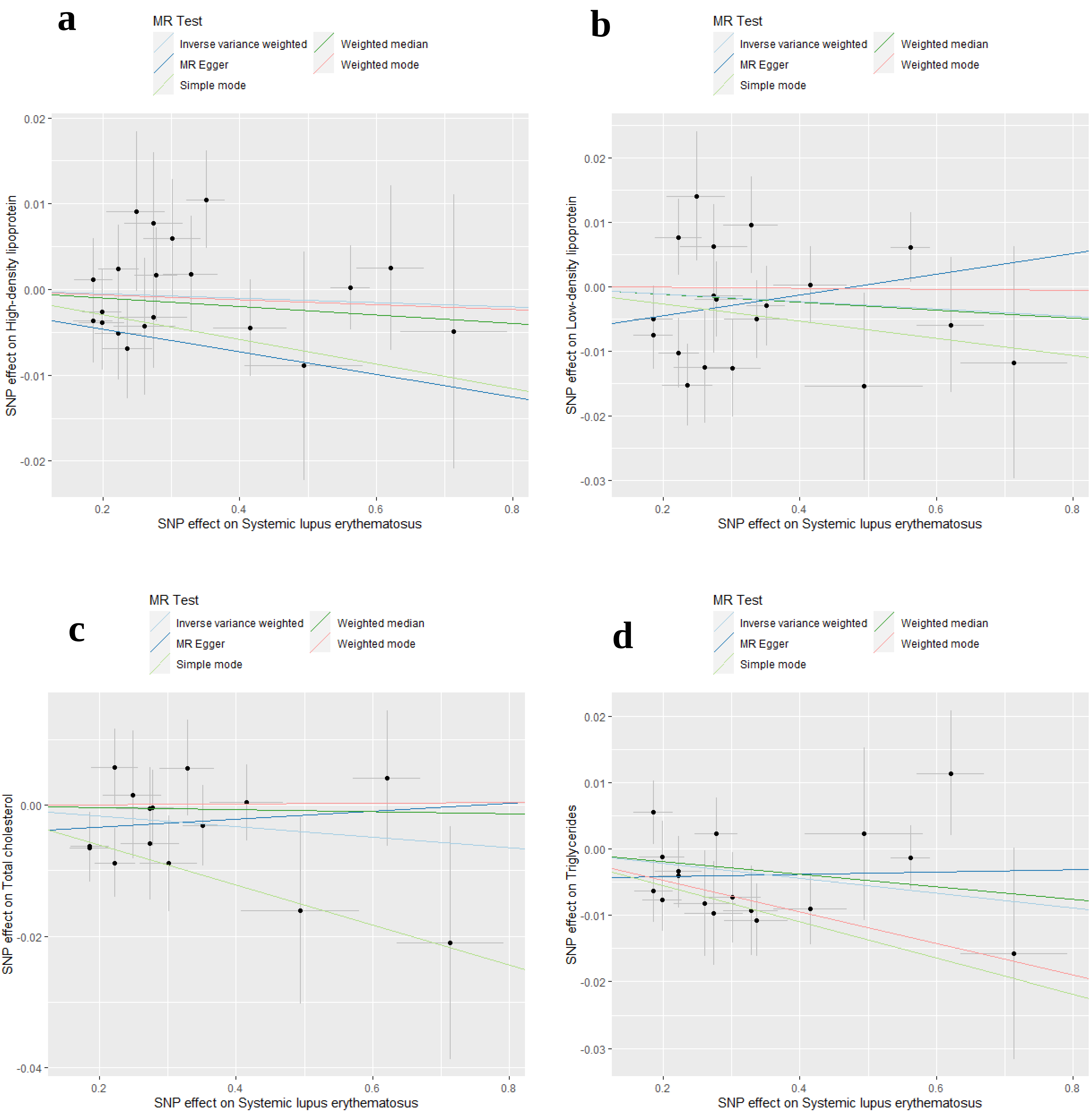
a
MR Test
Inverse variance weighted
MR Egger
Simple mode
Weighted median
Weighted mode
SNP effect on High-density lipoprotein
SNP effect on Systemic lupus erythematosus
b
MR Test
Inverse variance weighted
MR Egger
Simple mode
Weighted median
Weighted mode
SNP effect on Low-density lipoprotein
SNP effect on Systemic lupus erythematosus
c
MR Test
Inverse variance weighted
MR Egger
Simple mode
Weighted median
Weighted mode
SNP effect on Total cholesterol
SNP effect on Systemic lupus erythematosus
d
MR Test
Inverse variance weighted
MR Egger
Simple mode
Weighted median
Weighted mode
SNP effect on Triglycerides
SNP effect on Systemic lupus erythematosus
